# Comment on Haig et al. (2016): the conservation genetics juggling act: integrating genetics and ecology, science and policy

**DOI:** 10.1111/eva.12374

**Published:** 2016-05-17

**Authors:** Christian T. Smith, Brice Adams, Meredith Bartron, Mary K. Burnham‐Curtis, Emy Monroe, Jeffrey B. Olsen, Wade D. Wilson, Ashantye' Williams, Michael J. Millard, Molly A.H. Webb, John K. Wenburg

**Affiliations:** ^1^ Abernathy Fish Technology CenterU.S. Fish and Wildlife ServiceLongviewWAUSA; ^2^ Northeast Fishery CenterU.S. Fish and Wildlife ServiceLamarPAUSA; ^3^ Clark R. Bavin National Fish and Wildlife Forensic LaboratoryU.S. Fish and Wildlife ServiceAshlandORUSA; ^4^ Whitney Genetics LaboratoryU.S. Fish and Wildlife ServiceOnalaskaWIUSA; ^5^ Conservation Genetics LaboratoryU.S. Fish and Wildlife ServiceAnchorageAKUSA; ^6^ Southwestern Native Aquatic Resources and Recovery CenterU.S. Fish and Wildlife ServiceDexterNMUSA; ^7^ Conservation Genetics LaboratoryU.S. Fish and Wildlife ServiceWarm SpringsGAUSA; ^8^ Bozeman Fish Technology CenterU.S. Fish and Wildlife ServiceBozemanMTUSA

**Keywords:** applied research, U.S. Fish and Wildlife Service, Endangered Species Act

In their recent article, Haig et al. (2016) describe the genetic information needs of the U.S. Fish and Wildlife Service (Service) primarily for listing and recovery decisions related to the Endangered Species Act (ESA) and propose the creation of a National Center for Small Population Biology (NCSPB) to address them. We broadly agree with their description of information needs, but take issue with the implication that the Service does not already possess considerable scientific capacity to inform those decisions. As scientists who have spent substantial effort developing and providing scientific capacity within the Service, we feel compelled to provide some pertinent facts regarding that capacity. We are most familiar with the genetic capacity in the Service and as such that is our focus here, as it is in Haig et al. (2016). However, the Service also possesses significant internal expertise in most if not all of the other disciplines proposed in Haig et al. (2016) for the NCSPB (e.g., demographic analyses, pedigree analyses, population modeling, database management, sample curation, and policy and legal expertise; p. 191). We provide this comment to inform readers of Evolutionary Applications about our agency's scientific capacity and hope also it proves useful to those interested in evaluating the proposal for a NCSPB outlined by Haig et al. (2016).

Haig et al. (2016) do not explicitly state but certainly do imply that the Service does not have the types of internal scientific capacity they recommend for addressing ESA listing and recovery decisions. For instance, in reference to listing and recovery decisions, they state that ‘agencies and organizations hoping to find appropriate geneticists may need to perform time‐consuming searches when an issue arises, having a ready source of experts that could design and/or carry out research or consult about a species at risk would greatly facilitate this process’ (p.190), but do not mention the ready source of genetic expertise that currently resides in the Service. This is a major oversight we seek to remedy.

The Service first began to build conservation genetics capacity with the establishment of the Conservation Genetics Laboratory in Alaska in 1987 and development of a genetics facility at the National Fish and Wildlife Forensics Laboratory in Oregon in 1989. Genetic data played an increasing role in conservation management between the mid‐1990s and early 2000s, and the Service responded by creating additional applied conservation genetics facilities in its Fish Technology Centers in Washington State (Abernathy Fish Technology Center), Pennsylvania (Northeast Fishery Center), Georgia (Warm Springs Fish Technology Center), and New Mexico (Southwestern Native Aquatic Resources and Recovery Center). Most recently, in 2011, the Whitney Genetics Lab (now part of the Midwest Fisheries Center) was created in Wisconsin as a response to the invasion of non‐native carps and the growing need for analysis of environmental DNA (eDNA). Within these seven conservation genetics facilities (where the bulk of the Service genetics expertise exists), the agency employs more than 50 geneticists, more than 30 of which hold advanced degrees in disciplines relating to conservation genetics. Agency geneticists routinely address specific information needs of the Service and our partner agencies in a variety of ways, including ESA listing and recovery decisions, legal case reports, agency reports, reviews of external studies, consultations, and peer‐reviewed publications (e.g., see Appendix A for a list of over 130 peer‐reviewed papers with authors from Service genetic laboratories published since 2005).

Haig et al. (2016) reiterate the common call for ‘“bridging the gap” between the scientists who generate molecular data and conservation practitioners who use the data in a listing or status assessment or to establish recovery criteria and plan recovery actions’ (p.189). They cite the need for more molecular data and for agency personnel to understand how to apply those data to listing and recovery decisions. Further, they assert that ‘the types of investigations required for listing and recovery decisions are often not prized or rewarded in academia’ and that ‘researchers need to realize that the best listing and recovery decisions are made by integrating results from a number of data sets’ (p.189). We agree in general with their assertions, but note that they fail to point out that the Service has taken significant steps to address them; several examples follow.

Each of the seven Service conservation genetics facilities has a fully equipped genetics laboratory. These laboratories routinely generate the types of data mentioned by Haig et al. (2016; microsatellite and SNP genotyping, Sanger and next‐generation DNA sequencing) to address information needs of the Service (e.g., many of the publications in Appendix A). Haig et al. (2016) also rightly point out the need for systems and databases to organize, curate, and store data as well as samples, but again fail to mention the considerable resources the Service brings to bear on these issues. Proper storage and curation of both data and samples is a priority for Service laboratories to ensure maximum benefit and that the molecular data generated are available to the public. The Service's genetic laboratories have developed comprehensive storage facilities, systems and relational databases, and forensic experts at the Service's National Fish and Wildlife Forensics Laboratory insure that the collection and application of genetic data is appropriate and suitable for presentation in US courts.

Geneticists within the seven Service conservation genetics facilities routinely work with agency personnel to help them understand how genetic data apply to ESA listing and recovery issues. In addition to providing the results of their own research, agency geneticists provide expertise in a variety of other ways including serving on expert panels, providing meta‐analyses and helping develop study plans to ensure that research conducted internally or with partner laboratories efficiently addresses information needs specific to ESA listing and recovery decisions. In order to broaden access to the Service conservation genetics capacity, the agency also promotes a Conservation Genetics Community of Practice (www.fws.gov/ConservationGeneticsCOP/). This Community of Practice is a self‐directed interactive forum created to facilitate the exchange of information and technologies to strengthen the use and understanding of conservation genetics within the agency. It also promotes understanding of the application of genetic data to conservation management issues via classroom and online training (e.g., http://training.fws.gov/topic/online-training/webinars/advanced-conservation-genetics.html) and provides biologists with an initial contact and entry point to access the considerable genetic expertise within the Service.

We agree with Haig et al. (2016) that the types of studies needed for agency decisions related to the ESA are often not prized or rewarded in academia, highlighting a potential conflict for all researchers in applied sciences. Some agency information needs may be addressed by investigations that lead to novel or broadly applicable findings, facilitating publication of study results in the traditional peer‐reviewed literature. However, other needs require application of state‐of‐the‐art analyses and interpretation but are not expected to lead to novel or broadly applicable findings. This point highlights an important reason why the Service finds it advantageous to have internal scientific capacity. Performance measures and promotions for Service scientists are not linked directly to peer‐reviewed publications as they often are in academia and other government agencies. Similarly, Service scientific programs prioritize work based on agency needs rather than on the likelihood of the work leading to high‐impact publications. In fact, recognition of this issue was a major consideration in the Service's development of the Journal of Fish and Wildlife Management (www.fwspubs.org). The Service recognized the need to provide an outlet for applied studies that focused on the quality of the science and not necessarily the scale of inference, breadth of appeal or novelty of the work being published. As such, the overarching goal of the journal is not to achieve a high scientific impact factor, but rather to provide an outlet in which to publish rigorously peer‐reviewed applied science to meet the needs of the conservation community.

We also agree with Haig et al. (2016) that the best listing and recovery decisions integrate results from multiple data sets and disciplines. Moreover, the Service recognizes the value of multidisciplinary research and maintains applied research programs in fields like ecology, population modeling, fish passage, and physiology. These programs are similar to the conservation genetics program in that each employs experts in their respective fields and has access to field equipment, laboratories, and computational resources to carry out the mission of the agency. In most cases, these staff and assets are colocated with the conservation genetics infrastructure and work in close concert to integrate quantitative and ecological objectives in both ESA and non‐ESA issues.

We think that Haig et al. (2016) clearly articulated many of the scientific needs for ESA listing and recovery decisions. Further, we believe that this underscores why it is important for the Service to have internal scientific capacity in those key disciplines. The Service has created much of its scientific capacity based on the types of considerations noted by Haig et al. (2016), and maintains programs that do in fact address the seven objectives they list for their proposed NCSPB (p.190). Moreover, by integrating this scientific capacity within the agency, the Service facilitates close coordination not only among geneticists and managers, but also with scientists in other disciplines, agency solicitors, tribal liaisons, law enforcement professionals, and other specialists.

We would like to emphasize that we do not think that the Service scientific capacity is currently large enough to meet all of the agency's needs. For example, there is more need for genetic analyses and expertise related to ESA issues alone than current Service staff can address. In addition, we recognize that the work of existing Service genetics facilities has been (relative to all taxa listed under the ESA) disproportionately directed toward aquatic species, and not the taxonomic group that Haig et al. (2016) focused on in their article (birds). More importantly, we interpret the first phrase of the Service mission, ‘Working with others …’ to be an explicit acknowledgement that the rest of the statement ‘… to conserve, protect, and enhance fish, wildlife, plants, and their habitats for the continuing benefit of the American people’ is a larger goal than any one agency or group can achieve on its own. To this end, our agency routinely depends on partners (several of whom have built internal genetics capacity to address their own listing and recovery responsibilities) to help address genetic information needs. The vital nature of these partnerships is apparent in some of the case studies described by Haig et al. (2016). Work conducted within Service laboratories is similarly dependent on collaboration, and scientists representing cooperating agencies, tribes, universities, and public and private companies serve as coauthors on many of our reports and publications (e.g., many of the publications in Appendix A).

We agree with Haig et al. (2016) that bringing a variety of ‘scientific expertise under one umbrella group (perhaps virtual) would insure the accuracy in results, consistency across plans, appropriate interpretation of law and policy, and integration between research and non‐research biologists’ (p. 190). Moreover, we believe this is precisely why the Service should continue to improve upon and augment the considerable scientific capacity it currently maintains to inform decisions with the best information available.

## Supporting information


**Appendix A**. Peer‐reviewed publications produced by U.S. Fish and Wildlife genetics staff between 2005 and 2015.
