# Supplementary material for: Comment on Haig et al. (): the conservation genetics juggling act: integrating genetics and ecology, science and policy
Source: Evol Appl. 2016 May 17;9(5):635–7. doi: 10.1111/eva.12374 (PMC4869405; doi:10.1111/eva.12374)
Supplement: Supplementary file 1 — Appendix A. Peer‐reviewed publications produced by U.S. Fish and Wildlife genetics staff between 2005 and 2015. [file EVA-9-635-s001.docx]

**Appendix A.** Peer-reviewed publications produced by U.S. Fish and Wildlife genetics staff between 2005 and 2015.

Adams, B., R. Tabor, B. Thompson, P. W. DeHaan, and D. K. Hawkins 2013. Characterization of tetranucleotide microsatellite loci for Olympic mudminnow (*Novumbra hubbsi*). Conservation Genetics Resources **5**:573-575.

Amberg, J., S. McCalla, E. Monroe, R. Lance, K. Baerwaldt, and M. Gaikowski 2015. Improving efficiency and reliability of environmental DNA analysis for silver carp. Journal of Great Lakes Research **41**:367-373.

Araki, H., W. R. Ardren, E. Olsen, B. Cooper, and M. S. Blouin 2007. Reproductive success of captive-bred steelhead trout in the wild: Evaluation of three hatchery programs in the Hood River. Conservation Biology **21**:181-190.

Araki, H., R. S. Waples, W. R. Ardren, B. Cooper, and M. S. Blouin 2007. Effective population size of steelhead trout: influence of variance in reproductive success, hatchery programs, and genetic compensation between life-history forms. Molecular Ecology **16**:953-966.

Ardren, W. R., L. Amata, J. Whelan, and P. W. Dehaan 2007. Characterization of 16 highly variable tetranucleotide microsatellite loci for Oregon chub (Oregonichthys crameri) and cross amplification in Umpqua chub (O. kalawatseti). Molecular Ecology Notes **7**:808-810.

Ardren, W. R., J. Baumsteiger, and C. S. Allen 2010. Genetic analysis and uncertain taxonomic status of threatened Foskett Spring speckled dace. Conservation Genetics **11**:1299-1315.

Ardren, W. R., P. W. DeHaan, C. T. Smith, E. B. Taylor, R. Leary, C. C. Kozfkay, L. Godfrey, M. Diggs, W. Fredenberg, J. Chan, C. W. Kilpatrick, M. P. Small, and D. K. Hawkins 2011. Genetic structure, evolutionary history, and conservation units of bull trout in the coterminous United States. Transactions of the American Fisheries Society **140**:506-525.

Banks, M. A., D. P. Jacobson, I. Meusnier, C. A. Greig, V. K. Rashbrook, W. R. Ardren, C. T. Smith, J. Bernier-Latmani, J. Van Sickle, and K. G. O'Malley 2014. Testing advances in molecular discrimination among Chinook salmon life histories: evidence from a blind test. Animal Genetics **45**:412-420.

Baumsteiger, J., D. M. Hand, D. E. Olson, R. Spateholts, G. FitzGerald, and W. R. Ardren 2008. Use of parentage analysis to determine reproductive success of hatchery-origin spring chinook salmon outplanted into Shitike Creek, Oregon. North American Journal of Fisheries Management **28**:1472-1485.

Baumsteiger, J., D. Hankin, and E. J. Loudenslager 2005. Genetic analyses of juvenile steelhead, coastal cutthroat trout, and their hybrids differ substantially from field identifications. Transactions of the American Fisheries Society **134**:829-840.

Baumsteiger, J., and J. L. Kerby 2009. Effectiveness of salmon carcass tissue for use in DNA extraction and amplification in conservation genetic studies. North American Journal of Fisheries Management **29**:40-49.

Beacham, T. D., M. Wetklo, C. Wallace, J. B. Olsen, B. G. Flannery, J. K. Wenburg, W. D. Templin, A. Antonovich, and L. W. Seeb 2008. The application of microsatellites for stock identification of Yukon River Chinook salmon. North American Journal of Fisheries Management **28**:283-295. doi:10.1577/M06-253.1.

Bidon, T., A. Janke, S. R. Fain, H. G. Eiken, S. B. Hagen, U. Saarma, B. M. Hallstrom, N. Lecomte, and F. Hailer 2014. Brown and Polar bear Y chromosomes reveal extensive lame-biased gene flow within brother lineages. Molecular Biology and Evolution. **31**:1353-1363.

Bingham, D. M., B. M. Kennedy, K. C. Hanson, and C. T. Smith 2014. Loss of genetic integrity in hatchery steelhead produced by juvenile-based broodstock and wild integration: conflicts in production and conservation goals. North American Journal of Fisheries Management **34**:609-620.

Bond, M. H., P. A. Crane, W. A. Larson, and T. P. Quinn 2014. Is isolation by adaptation driving genetic divergence among proximate Dolly Varden char populations? Ecology and Evolution **4**:2515-2532.

Braaten, P. J., D. B. Fuller, R. D. Lott, T. M. Haddix, L. D. Holte, R. H. Wilson, M. L. Bartron, J. A. Kalie, P. W. DeHaan, W. Ardren, R. J. Holm, and M. E. Jaeger 2012. Natural growth and diet of known-age pallid sturgeon (Scaphirhynchus albus) early life stages in the upper Missouri River basin, Montana and North Dakota. Journal of Applied Ichthyology **28**:496-504.

Bromaghin, J. F., and P. A. Crane 2005. A method to bin alleles of genetic loci that maintains population heterogeneity. Canadian Journal of Fisheries and Aquatic Sciences **62**:1570-1579.

Bromaghin, J. F., D. F. Evenson, T. H. McLain, and B. G. Flannery 2011. Using a genetic mixture model to study phenotypic traits: differential fecundity among Yukon River Chinook salmon. Transactions of the American Fisheries Society **140**:235-249. doi:10.1080/00028487.2011.558776.

Brown, S. K., J. M. Hull, D. R. Updike, S. R. Fain, and H. B. Ernest 2009. Black bear population genetics in California: Signatures of population structure, competitive release, and historical translocation. Journal of Mammology **193**:1066-1074.

Budowle, B., P. Garofano, A. Hellman, M. Ketchum, S. Kanthaswamy, W. Parson, W. van Haeringen, S. Fain, and T. Broad 2005. Recommendations for animal DNA Forensic and identity testing. International Journal of Legal Medicine **119**:295-302.

Burnham-Curtis, M. K., P. W. Trail, R. Kagan, and M. K. Moore 2015. Wildlife Forensics: An overview and update for the prosecutor. United States Attorney’s Bulletin. May 2015. Pp 53-68.

Campton, D. E. 2005. Sperm competition in salmon hatcheries - The need to institutionalize genetically benign spawning protocols: Response to comment. Transactions of the American Fisheries Society **134**:1495-1498.

Campton, D. E., and L. R. Kaeding 2005. Westslope cutthroat trout, hybridization, and the US Endangered Species Act. Conservation Biology **19**:1323-1325.

Cavileer, T. D., S. S. Hunter, J. Olsen, J. Wenburg, and J. J. Nagler 2015. A sex determining gene (sdY) assay shows discordance between phenotypic and genotypic sex in wild populations of Chinook salmon. Transactions of the American Fisheries Society **144**:423-430. doi:0.1080/00028487.2014.993479.

Chambers, S. M., S. R. Fain, B. Fazio, and M. Amaral 2012. An account of the taxomony of North American wolves from morphological and genetic analyses. North American Fauna. **77**:1-67.

Chen, Y., C. Conway, C. Keeler-Foster, R. Hamman, and S. Meismer 2009. Genetic characterization of variation in captive and wild woundfin. North American Journal of Fisheries Management **29**:843-849.

Chen, Y., M. R. Childs, and C.Keeler-Foster 2011. Evaluation of woundfin augmentation efforts in the Virgin River by estimation of admixture proportions. Transactions of the American Fisheries Society **140**:598-604.

Cook, G. M., J. P. Rothenberger, M. Sikaroodi, P. M. Gillevet, E. C. Peters, and R. B. Jonas 2013. A comparison of culture-dependent and culture-independent techniques used to characterize bacterial communities on healthy and white plague-diseased corals of the Montastraea annularis species complex. Coral Reefs **32**:375-388. doi:10.1007/s00338-012-0989-6.

Crane, P., P. Walsh, C. Lewis, and J. K. Wenburg 2015. Origin and genetic diversity of lake trout in the Togiak National Wildlife Refuge, Alaska. Journal of Fish and Wildlife Management **6**:130-144; e1944-687X. doi:10.3996/032014-JFWM-022.

Daniels, E., and G. Cook 2012. Coral Triangle Seascapes. Asian Geographic LTD., Singapore. 131 pp. ISBN 10: 9810718551; ISBN 13: 9789810718558.

Daum, D. W., and B. G. Flannery 2011. Canadian-origin Chinook salmon rearing in non-natal U.S. tributary streams of the Yukon River, Alaska. Transactions of the American Fisheries Society **140**:207-220. doi:10.1080/00028487.2011.545004.

Davis, L. A., T. Wagner, M. L. Bartron. 2015. Spatial and temporal movement dynamics of brook Salvelinus fontinalis and brown trout Salmo trutta. Environmental Biology of Fishes 98: 2049-2065.

DeHaan, P. W., B. A. Adams, R. A. Tabor, D. K. Hawkins and B. Thompson 2014. Historical and contemporary forces shape genetic variation in the Olympic mudminnow (*Novumbra hubbsi*), an endemic fish from Washington State, USA. Conservation Genetics **15**:1417-1431.

DeHaan, P. W., and W. R. Ardren 2005. Characterization of 20 highly variable tetranucleotide microsatellite loci for bull trout (*Salvelinus confluentus*) and cross-amplification in other Salvelinus species. Molecular Ecology Notes **5**:582-585.

DeHaan, P. W., C. A. Barfoot, and W. R. Ardren 2007. Genetic analysis of bull trout populations on the Flathead Indian Reservation, Montana. Paper read at Wild Trout Symposium IX - Sustaining Wild Trout in a Changing World (2007).

DeHaan, P. W., S. B. Bernall, J. M. DosSantos, L. L. Lockard, and W. Ardren 2011. Use of genetic markers to aid in re-establishing migratory connectivity in a fragmented metapopulation of bull trout (*Salvelinus confluentus*). Canadian Journal of Fisheries and Aquatic Sciences **68**:1952-1969.

DeHaan, P. W., and S. R. Bernall 2013. Spawning Success of Bull Trout Transported Above Mainstem Clark Fork River Dams in Idaho and Montana. North American Journal of Fisheries Management **33**:1269-1282.

DeHaan, P. W., S. J. Brenkman, B. Adams, and P. Crain 2011. Genetic population structure of Olympic Peninsula bull trout populations and implications for Elwha Dam removal. Northwest Science **85**:463-475.

DeHaan, P. W., G. R. Jordan, and W. R. Ardren 2008. Use of genetic tags to identify captive-bred pallid sturgeon (*Scaphirhynchus albus*) in the wild: improving abundance estimates for an endangered species. Conservation Genetics **9**:691-697.

DeHaan, P. W., S. V. Libants, R. F. Elliott, and K. T. Scribner 2006. Genetic population structure of remnant lake sturgeon populations in the upper Great Lakes basin. Transactions of the American Fisheries Society **135**:1478-1492.

DeHaan, P. W., C. Pascal, and J. E. Seeb 2014. Novel SNP genotyping assays facilitate species identification of *Salvelinus* collected in a recreational fishery. North American Journal of Fisheries Management **143**:164–172.

DeHaan, P. W., P. D. Scheerer, R. Rhew, and W. Ardren 2012. Analysis of genetic variation in populations of Oregon Chub, a threatened floodplain minnow in a highly altered envrionment. Transactions of the American Fisheries Society **141**:533-549.

DeHaan, P. W., L. T. Schwabe, and W. R. Ardren 2010. Spatial patterns of hybridization between bull trout, *Salvelinus confluentus*, and brook trout, *Salvelinus fontinalis* in an Oregon stream network. Conservation Genetics **11**:935-949.

Díaz-Ferguson, E, A. S. Williams, and G. R. Moyer 2011. Isolation and characterization of microsatellite loci for the fat pocketbook mussel (*Potamilus capax*). Molecular Ecology Resources. **11**:219–222.

Diaz-Ferguson, A. S. Williams, and G. R. Moyer 2011. Isolation and characterization of microsatellite loci for the federally endangered fat threeridge mussel (*Amblema neislerii*). Conservation Genetic Resources **3**: 757-759.

Diaz-Ferguson, E., J. Herod, J. Galvez, and G. R. Moyer 2014. Development of Molecular Markers for eDNA Detection of the Invasive African jewelfish (*Hemichromis letourneuxi*): A New Tool for Monitoring Aquatic Invasive Species in National Wildlife Refuges. Management of Biological Invasions.**5**:121-131.

Diaz-Ferguson, E., and G. R. Moyer 2014. History, applications, methodological issues and perspectives for the use environmental DNA (eDNA) in marine and freshwater environments. International Journal of Tropical Biology and Conservation. **62**: 1273-1284.

Diggs, M. D., and W. R. Ardren 2008. Characterization of 12 highly variable tetranucleotide microsatellite loci for Arctic grayling (Thymallus arcticus) and cross amplification in other Thymallus species. Molecular Ecology Resources **8**:828-830.

Echelle, A. A., M. de Lourdes Lozano Vilano, S. Baker, W. D. Wilson, A. F. Echelle, G. P. Garrett, and R. J. Edwards 2013. Conservation Genetics of Gambusia krumholzi (Teleostei: Poeciliidae) with Assessment of the Species Status of G. clarkhubbsi and Hybridization with G. speciosa. Copeia **2013**:72-79.

Fain, S. R., D. J. Straughan, and B. F. Taylor 2010. Genetic outcomes of wolf recovery in the western Great Lakes states. Conservation Genetics. **11**:1747-1765.

Fain, S. R., D. J.Straughan, B. C. Hamlin, R. M. Hoesch, and J. P. LeMay 2013. Forensic genetic identification of sturgeon caviars traveling in world trade. Conservation Genetics. **14**:855-874.

Ferriera, J. M., M. Burnham-Curtis, F. Schunck, M. Rocha, C. Miyaki, L. F. Silveira, M. Melo, and J. S. Morgante 2014. Development of polymorphic microsatellite markers for four bird species exploited by the illegal wildlife trade in Brazil. Conservation Genetics Resources **7**:435-436. doi:10.1007/s12686-014-0389-z.

Flannery, B. G., T. D. Beacham, J. R. Candy, R. R. Holder, G. F. Maschmann, E. J. Kretschmer, and J. K. Wenburg 2010. Mixed-Stock Analysis of Yukon River chum salmon: application and validation in a complex fishery. North American Journal of Fisheries Management **30**:1324-1338.

Flannery, B. G., P. A. Crane, J. H. Eiler, T. D. Beacham, N. A. Decovich, W. D. Templin, O. L. Schlei, and J. K. Wenburg JK 2012. Comparison of radiotelemetry and microsatellites for determining the origin of Yukon River Chinook salmon. North American Journal of Fisheries Management **32**:720-730.

Flannery, B. G., R. E. Spangler, B. L. Norcross, C. J. Lewis, and J. K. Wenburg 2013. Microsatellite analysis of population structure in Alaska eulachon with application to mixed-stock analysis. Transactions of the American Fisheries Society **142**:1036-1048.

Flannery, B. G., J. K. Wenburg, and A. J. Gharrett 2007. Evolution of mitochondrial DNA variation within and among Yukon River chum salmon populations. Transactions of the American Fisheries Society **136**:902-910.

Flannery, B. G., J. K. Wenburg, A. J. Gharrett 2007. Variation of amplified fragment length polymorphisms in Yukon River chum salmon: population structure and application to mixed-stock analysis. Transactions of the American Fisheries Society **136**:911-925.

Galindo, R., W. D. Wilson, and C. Caldwell. in press. Geographic Distribution of Genetic Diversity in Populations of Rio Grande Chub (*Gila pandora*). Conservation Genetics.

Garner, B. A., B. K. Hand, S. J. Amish, L. Bernatchez, J. T. Foster, K. M. Miller, P. A. Morin, S. R. Narum, S. J. O’Brien, G. Roffler, W. D. Templin, P. Sunnucks, J. Strait, K. I. Warheit, T. R. Seamons, J. K. Wenburg, J. Olsen, G. Luikart (in press) Genomics in Conservation: Case Studies and Bridging the Gap between Data and Application. Trends in Ecology and Evolution. doi: http://dx.doi.org/10.1016/j.tree.2015.10.009.

Gharrett, A. J., A. P. Matala, E. L. Peterson, A. K. Gray, Z. Z. Li, and J. Heifetz 2005. Two genetically distinct forms of rougheye rockfish are different species. Transactions of the American Fisheries Society **134**:242-260.

Gomez-Uchida, D., J. E. Seeb, M. J. Smith, C. Habicht, T. P. Quinn, and L. W. Seeb 2011. Single nucleotide polymorphisms unravel hierarchical divergence and signatures of selection among Alaskan sockeye salmon (*Oncorhynchus nerka*) populations. Bmc Evolutionary Biology **11**:48.

Guinand, B., K. S. Page, M. K. Burnham-Curtis, and K. T. Scribner 2012. Genetic signatures of historical bottlenecks in sympatric lake trout (*Salvelinus namaycush*) morphotypes in Lake Superior. Environmental Biology of Fishes. **95**:323-334.

Guinand, B., K. T. Scribner, K. S. Page, K. Filcek, L. Main, and M. K. Burnham-Curtis 2005. Effects of Coancestry on Accuracy of Individual Assignments to Population of Origin: Examples Using Great Lakes Lake Trout (*Salvelinus namaycush*). Genetica. **127**: 329-340.

Hailer, F., V. E. Kutcshera, B. M. Hallstrom, D. Klassert, S. R. Fain, J. A. Leonard, U. Arnason, and A. Janke 2012. Nucelar genomic sequences reveal that Polar bears are an old and distinct bear lineage. Science. **336**:344-347.

Hard, J. J., D. G. Elliott, R. J. Pascho, D. M. Chase, L. K. Park, J. R. Winton, and D. E. Campton 2006. Genetic effects of ELISA-based segregation for control of bacterial kidney disease in Chinook salmon (*Oncorhynchus tshawytscha*). Canadian Journal of Fisheries and Aquatic Sciences **63**:2793-2808.

Hard, J. J., D. G. Elliott, R. J. Pascho, D. M. Chase, L. K. Park, J. R. Winton, and D. E. Campton 2007. Potential trade-offs in resistance of chinook salmon (*Oncorhynchus tshawyacha*) to two bacterial pathogens resulting from selection of broodstock based on antigen level. Aquaculture **272**:S267-S267.

Inoue, K., G. R. Moyer, A. S. Williams, W. Monroe, and D. Berg 2011. Isolation and characterization of 17 polymorphic microsatellite loci in the spectaclecase, *Cumberlandia monodonta* (Bivalvia: Margaritiferidae). Conservation Genetics Resources **3**:57-60.

Julian, S.E., M. L. Bartron, and J. A. Kalie. 2014. Tri- and tetra-nucleotide microsatellite DNA markers for the rayed bean freshwater mussel (Villosa fabalis). Conservation Genetics Resources 6: 613-615.

Julian, S. E., and M. L. Bartron. 2007. Microsatellite markers for American shad (Alosa sapidissima) and cross-species amplification within the family Clupeidae. Molecular Ecology Notes 7: 805-807.

Johnson, J. R., J. Baumsteiger, J. Zydlewski, J. M. Hudson, and W. Ardren 2010. Evidence of panmixia between sympatric life history forms of coastal cutthroat trout in two lower Columbia River tributaries. North American Journal of Fisheries Management **30**:691-701.

Jorstad, K. E., C. T. Smith, Z. Grauvogel, and L. Seeb 2007. The genetic variability of the red king crab, *Paralithodes camtschatica* (Tilesius, 1815) (Anomura, Lithodidae) introduced into the Barents Sea compared with samples from the Bering Sea and Kamchatka region using eleven microsatellite loci. Hydrobiologia **590**:115-121.

Kassler, T. W., D. K. Hawkins, and J. M. Tipping 2008. Summer-run Hatchery steelhead have naturalized in the South Fork Skykomish River, Washington. Transactions of the American Fisheries Society **137**:763-771.

Kretschmer E. J., J. B. Olsen, and J. K. Wenburg 2009. Characterization of eight microsatellite loci in Sea Otter, *Enhydra lutris*, and cross-species amplification in other Mustelidae. Conservation Genetics **10**:775-777. doi:10.1007/s10592-008-9660-8

Kutschera, V., T. Bidon, F. Hailer, J. L. Rodi, S. R. Fain, and A. Janke 2014. Bears in a foreset of gene trees: Phylogenetic inference is complicated by incomplete lineage sorting and gene flow. Molecular Biology and Evolution. **31**:2004-2017.

Lacroix, G. L., D. Knox, T. F. Sheehan, M. D. Renkawitz, and M. L. Bartron 2012. Distribution of U.S. Atlantic salmon postsmolts in the Gulf of Maine. Transactions of the American Fisheries Society **141**:934-942.

Lemons P. R., T. C. Marshall, S. E. McCloskey, S. A. Sethi, J. A. Schmutz, and J. S. Sedinger 2015. A new likelihood-based approach for assessment of extra-pair paternity and conspecific brood parasitism in natural populations. Molecular Ecology Resources **15**:107-116.

Martin, A. P., A. A. Echelle, G. Zegers, S. Baker, and C. L. Keeler-Foster 2012. Dramatic shifts in the gene pool of a managed population of an endangered species may be exacerbated by high genetic load. Conservation Genetics **13**:349-358.

Martin, R. M., C. L. Keeler-Foster, K. G. Boykin, G. Zegers, and W. D. Wilson 2012. Isolation and characterization of eight novel microsatellite loci in endangered Wyoming toad, *Bufo baxteri*. Conservation Genetics Resources **4**:347-349.

Martin, R. M., M. L. Robinson, and W. D. Wilson 2015. Isolation and characterization of twenty-five novel microsatellite loci in Colorado pikeminnow, *Ptychocheilus lucius*, with cross-species amplification for eight other cyprinids. Conservation Genetics Resources **7**:113-117.

Matala, A. P., R. French, E. Olsen, and W. R. Ardren 2009. Ecotype distinctions among Steelhead in Hood River, Oregon, allow real-time genetic assignment of conservation broodstocks. Transactions of the American Fisheries Society **138**:1490-1509.

Matala, A. P., S. Marx, and T. G. Wise 2008. A genetically distinct wild redband trout (*Oncorhynchus mykiss gairdneri*) population in Crane Prairie Reservoir, Oregon, persists despite extensive stocking of hatchery rainbow trout (*O-m. irideus*). Conservation Genetics **9**:1643-1652.

McGlauflin, M. T., D. E. Schindler, L. W. Seeb, C. T. Smith, C. Habicht, and J. E. Seeb 2011. Spawning habitat and geography influence population structure and juvenile migration timing of sockeye salmon in the Wood River Lakes, Alaska. Transactions of the American Fisheries Society **140**:763-782.

McPhee M. V., M. S. Zimmerman, T. D. Beacham, B. R. Beckman, J. B. Olsen, L. W. Seeb, and W. D. Templin 2009. A hierarchical framework to identify influences on Pacific salmon population abundance and structure in the Arctic-Yukon-Kuskokwim region. In: Pacific Salmon: Ecology and Management of Western Alaska’s Populations (eds. Krueger CC, Zimmerman CE), pp. 1177-1198. American Fisheries Society Symposium 70. Bethesda, Maryland.

Millard, M. J., D. R. Smith, E. Obert, J. Grazio, M. L. Bartron, C. Wellington, S. Grise, S. Rafferty, R. Wellington, & S. Julian. 2009. Movements of Brown Bullhead in Presque Isle Bay, Lake Erie, Pennsylvania. Journal of Great Lakes Research 35: 613-619.Monroe, E., and H. Britten 2014. Conservation in Hine’s sight: the conservation genetics of the federally endangered Hine’s Emerald Dragonfly, *Somatochlora hineana*. Journal of Insect Conservation **18**:353-363.

Monroe, E., and H. Britten 2015. Single-sample estimation of effective population size in several populations of the endangered Hine’s emerald dragonfly. Freshwater Science **34**:1058-1064.

Moran, P., D. J. Teel, M. A. Banks, T. D. Beacham, M. R. Bellinger, S. M. Blankenship, J. R. Candy, J. C. Garza, J. E. Hess, S. R. Narum, L. W. Seeb, W. D. Templin, C. G. Wallace, and C. T. Smith 2013. Divergent life-history races do not represent Chinook salmon coast-wide: the importance of scale in Quaternary biogeography. Canadian Journal of Fisheries and Aquatic Sciences **70**:415-435.

Moyer, G. R., B. L. Sloss, B. R. Kreiser, and K. A. Feldheim 2009. Isolation and characterization of microsatellite loci for alligator gar (*Atractosteus spatula*) and their variability in two other species (*Lepisosteus oculatus* and *L. osseus*) of Lepisosteidae. Molecular Ecology Resources **9**:1-4.

Moyer, G. R., J. D. Rousey, and M. Cantrell 2009. Evaluation of genetic diversity and relatedness of hatchery and wild sicklefin redhorse (*Moxostoma* sp.) of the Little Tennessee River. North American Journal of Fisheries Management **29**:1438-1443.

Moyer, G. R., and A. S. Williams 2011. Isolation and characterization of microsatellite loci for the oval pigtoe mussel, *Pleurobema pyriforme* (Lea 1857). Conservation Genetic Resources **3**:255-257.

Moyer, G. R., J. Sweka, and D. Peterson 2012. Past and present processes influencing genetic diversity and effective population size in a natural population of Atlantic sturgeon, *Acipenser oxyrinchus oxyrinchus*. Transactions of the American Fisheries Society **141**:56-67.

Moyer, G. R., and E. Diaz-Ferguson 2012. Identification of endangered Alabama lampmussel (*Lampsilis virescens*) specimens collected in the Emory River, Tennessee, USA via DNA barcoding. Conservation Genetics **13**:885-889.

Moyer G. R., and A. S. Williams 2012. Assessment of genetic diversity for American shad in the Santee-Cooper river basin of South Carolina prior to hatchery augmentation. Marine Coastal Fisheries **4**:312-26.

Moyer, G. R, A. S. Williams, M. L. Jolley, B. J. Ragland, and T. N. Churchill 2014. Genetic confirmation and assessment of an unauthorized fish introduction in Parksville Reservoir, Tennessee. **Journal of the Southeastern Association of Fish and Wildlife Agencie**s. **1**:64-69.

Moyer, G. R, A. George, P. Rakes, J. Shute, and A. S. Williams 2015. Assessment of genetic diversity and hybridization for the endangered Conasauga logperch (*Percina jenkinsi*). Southeastern Fishes Council Proceedings. (MS # 1059).

Oleinik, A. G., L. A. Skurikhina, V. A. Brykov, P. A. Crane, and J. K. Wenburg 2005. Differentiation of Dolly Varden char *Salvelinus malma* from Asia and North America inferred from PCR-RFLP analysis of mitochondrial DNA. Russian Journal of Genetics **41**:501-508.

Olsen, J. B., T. D. Beacham, M. Wetklo, L. W. Seeb, C. T. Smith, B. G. Flannery, and J. K. Wenburg 2010. The influence of hydrology and waterway distance on population structure of Chinook salmon *Oncorhynchus tshawytscha* (Walbaum) in a large river. Journal of Fish Biology **76**:1128-1148. doi:10.1111/j.1095-8649.2010.02554.x

Olsen, J. B., P. A. Crane, B. G. Flannery, K. Dunmall, W. D. Templin, and J. K. Wenburg 2011. Comparative landscape genetics of three Pacific salmon species from subarctic North America. Conservation Genetics. **12**:223-241. doi:10.1007/s10592-010-0135-3.

Olsen, J. B., B. G. Flannery, T. D. Beacham, J. B. Bromaghin, P. A. Crane, C. F. Lean, K. M. Dunmall, and J. K. Wenburg 2008. The influence of hydrographic structure and seasonal run timing on genetic diversity and isolation-by-distance in chum salmon (Oncorhynchus keta). Canadian Journal of Fisheries and Aquatic Sciences **65**:2026-2042.

Olsen, J. B., C. J. Lewis, R. L. Massengill, K. J. Dunker, and J. K. Wenburg 2015. An evaluation of target specificity and sensitivity of three qPCR assays for detecting environmental DNA from Northern Pike (*Esox lucius*). Conservation Genetics Resources **7**:615-617. doi:10.1007/s12686-015-0459-x.

Olsen, J. B., S. J. Miller, K. Harper, J. J. Nagler, and J. K. Wenburg 2006. Contrasting sex ratios in juvenile and adult Chinook salmon (*Oncorhynchus tshawytscha*) from southwest Alaska: sex reversal or differential survival? Journal of Fish Biology **69(sa)**:140-144.

Olsen, J. B., S. J. Miller, K. Harper, and J. K. Wenburg 2009. Genetic health and variables influencing the effective number of breeders in western Alaska Chinook salmon. In: Pacific Salmon: Ecology and Management of Western Alaska’s Populations (eds. Krueger CC, Zimmerman CE), pp. 781-795. American Fisheries Society Symposium 70. Bethesda, Maryland.

Olsen, J. B., K. Wuttig, D. Fleming, E. J. Kretschmer, and J. K. Wenburg 2006. Evidence of partial anadromy and resident-form dispersal bias on a fine scale in populations of *Oncorhynchus mykiss*. Conservation Genetics **7**:613-619.

Page, K. S., K. T. Scribner, D. Bast, M. E. Holey, and M. K. Burnham-Curtis 2005. Genetic evaluation of a Great Lakes lake trout hatchery program. Transactions of the American Fisheries Society. **134**:872-891.

Peterson, D. P., and W. R. Ardren 2009. Ancestry, population structure, and conservation genetics of Arctic grayling (*Thymallus arcticus*) in the upper Missouri River, USA. Canadian Journal of Fisheries and Aquatic Sciences **66**:1758-1774.

Pine III, W. E., B. Healy, E. O. Smith, M. Trammell, D. Speas, R. Valdez, M. Yard, C. Walters, R. Ahrens, R. Van Haverbeke, D. Stone, and W. Wilson 2013. An individual-based model for population viability analysis of humpback chub in Grand Canyon. North American Journal of Fisheries Management, **33**:626-641.

Reimchen, T. E., R. J. Nelson, and C. T. Smith 2008. Estimating deer colonization rates to offshore islands of Haida Gwaii, British Columbia, using microsatellite markers. Paper read at Lessons from the Islands.

Robinson, J. D., J. W. Simmons, A. S. Williams, and G.R. Moyer 2013. Population structure and genetic diversity in the endangered bluemask darter (*Etheostoma akatulo*). Conservation Genetics **14**:79-92.

Robinson, J., and G. R. Moyer 2013. Linkage disequilibrium and effective population size when generations overlap. Evolutionary Applications **6**:290-302.

Ruff, C. P., D. E. Schindler, J. B. Armstrong, K. T. Bentley, G. T. Brooks, G. W. Holtgrieve, M. T. McGlauflin, C. E. Torgerson, and J. E. Seeb 2011. Temperature-associated population diversity in salmon confers benefits to mobile consumers. Ecology **92**:2073-2084.

Schlei, O. L., A. Crête-Lafrenière, A. R. Whiteley, R. J. Brown, J. B. Olsen, L. Bernatchez, and J. K. Wenburg 2008. DNA barcoding of eight North American coregonine species. Molecular Ecology Resources **8**:1212-1218. doi:10.1111/j.1755-0998.2008.02350.x.

Seeb, L. W., A. Antonovich, A. A. Banks, T. D. Beacham, A. R. Bellinger, S. M. Blankenship, A. R. Campbell, N. A. Decovich, J. C. Garza, C. M. Guthrie, T. A. Lundrigan, P. Moran, S. R. Narum, J. J. Stephenson, K. J. Supernault, D. J. Teel, W. D. Templin, J. K. Wenburg, S. E. Young, and C. T. Smith 2007. Development of a standardized DNA database for Chinook salmon. Fisheries **32**:540-552.

Sethi, S. A., and E. Benolkin 2013. Detection efficiency and habitat use to inform inventory and monitoring efforts: juvenile coho salmon in the Knik River basin. Alaska. Ecology of Freshwater Fish **22**:398-411. doi:10.1111/eff.12034

Sethi, S. A., G. M. Cook, P. Lemons, and J. K. Wenburg 2014. Guidelines for MSAT and SNP panels that lead to high quality data for genetic mark recapture studies. Canadian Journal of Zoology **92**:515-526.

Sethi, S. A., and T. Tanner 2013. Bayesian implementation of a time stratified Lincoln-Petersen estimator for salmon abundance in the Matanuska River, Alaska, USA. Fisheries Research **145**:90-99. doi:10.1016/j.fishres.2013.02.004.

Sethi, S. A., and T. L. Tanner 2014. Spawning distribution and abundance of a northern Chinook population. Fisheries Management and Ecology **21**:427-438.

Sethi, S. A., and M. Dalton 2012. Risk measures for natural resource management: description, simulation testing and R code with fisheries examples. Journal of Fish and Wildlife Management **3**:150-157.

Sethi, S. A., M. Dalton, and R. Hilborn 2012. Managing harvest risk with catch pooling cooperatives. ICES Journal of Marine Science **69**:1038-1044.

Sethi, S. A., M. Dalton, and R. Hilborn 2012. Quantitative risk measures applied to Alaskan commercial fisheries. Canadian Journal of Fisheries and Aquatic Sciences **69**:487-498.

Shedd, K. R., F. A. von Hippel, J. J. Willacker, T. R. Hamon, O. L. Schlei, J. K. Wenburg, J. L. Miller, and S. A. Pavey 2015. Ecological release leads to novel ontogenetic diet shift in kokanee (*Oncorhynchus nerka*). Canadian Journal of Fisheries and Aquatic Sciences **72**:1718-1730. doi:10.1139/cjfas-2015-0146

Smith, C. T., A. Antonovich, W. D. Templin, C. M. Elfstrom, S. R. Narum, and L. W. Seeb 2007. Impacts of marker class bias relative to locus-specific variability on population inferences in Chinook salmon: A comparison of single-nucleotide polymorphisms with short tandem repeats and allozymes. Transactions of the American Fisheries Society **136**:1674-1687.

Smith, C. T., J. Baumsteiger, W. R. Ardren, Y. Dettlaff, D. Hawkins, and D. M. Van Doornik 2015. Eliminating variation in age-at-spawning leads to genetic divergence within a single coho salmon population. Journal of Fish and Wildlife Management **6**:4-18.

Smith, C. T., and R. Engle 2011. Persistent reproductive isolation between sympatric lineages of fall Chinook salmon in White Salmon River, Washington. Transactions of the American Fisheries Society **140**:699-715.

Smith, C. T., R. French, J. Lovtang, and D. Hand 2014. Genetic Composition of the Warm Springs River Chinook Salmon Population Maintained following Eight Generations of Hatchery Production. Transactions of the American Fisheries Society **143**:1280-1294.

Smith, C. T., S. B. Reid, L. Godfrey, and W. R. Ardren 2011. Gene flow among Modoc sucker and Sacramento sucker populations in the upper Pit River. Journal of Fish and Wildlife Management **2**:72-84.

Smith, C. T., and L. W. Seeb 2008. Number of alleles as a predictor of the relative assignment accuracy of short tandem repeat (STR) and single-nucleotide-polymorphism (SNP) baselines for chum salmon. Transactions of the American Fisheries Society **137**:751-762.

Smith, M. J., C. E. Pascal, Z. Grauvogel, C. Habicht, J. E. Seeb, and L. W. Seeb 2011. Multiplex preamplification PCR and microsatellite validation enables accurate single nucleotide polymorphism genotyping of historical fish scales. Molecular Ecology Resources **11**:268-277.

Stephenson, J. J., M. R. Campbell, J. E. Hess, C. Kozfkay, A. P. Matala, M. V. McPhee, P. Moran, S. R. Narum, M. M. Paquin, O. Schlei, M. P. Small, D. M. Van Doornik, and J. K. Wenburg 2009. A centralized model for creating shared, standardized, microsatellite data that simplifies inter-laboratory collaboration. Conservation Genetics **10**:1145-1149.

Sutherland, B. J. G., K. C. Hanson, J. R. Jantzen, B. F. Koop, and C. T. Smith 2014. Divergent immunity and energetic programs in the gills of migratory and resident *Oncorhynchus mykiss*. Molecular Ecology **23**:1952-1964.

Sykes, C. L. and W. D. Wilson 2015. Challenges of Developing a Chemical Method for Removing Quagga Mussel Veligers from Fish Transport Tanks. In, Biology and Managemetn of Invasive Quagga and Zebra Mussels in the Western United States (pp. 487-506). CRC Press.

Teel, D. J., S. R. Narum, J. B. Olsen, and F. M. Utter 2011. Introduction to a special section: Genetic adaptation of natural salmonid populations. Transactions of the American Fisheries Society **140**:659-664. doi:10.1080/00028487.2011.583537.

Von Bargen, J., J. Rueth, and C. T. Smith 2015. Development of a Chinook Salmon Sex Identification SNP Assay based on the Growth Hormone Pseudogene. Journal of Fish and Wildlife Management **6**:213-219.

Vu, N. V., C. L. Keeler‐Foster, I. B. Spies, and D. T. Ribeiro 2005. Twelve microsatellite markers developed in woundfin (*Plagopterus argentissimus*), an endangered warmwater fish of the lower Colorado River basin. Molecular Ecology Notes **5**:302-304.

Wang, R., C. Li, J. A. Stoeckel, G. Moyer, Z. Liu, and E. Peatman 2012. Rapid development of molecular resources for a freshwater mussel, *Villosa lienosa* (Bivalvia: Unionidae) using a RNA-seq-based approach. Freshwater Science **31**:695-708.

Whiteley, A. R., K. Hastings, J. K. Wenburg, C. A. Frissell, J. C. Martin, and F. W. Allendorf 2010. Genetic variation and effective population size in isolated populations of coastal cutthroat trout. Conservation Genetics. **11**:1929-1943. doi:10.1007.s10592-010-0083-y.

Williams, A. S., and G. R. Moyer 2012. Isolation and characterization of 21 microsatellite loci for the federally threatened yellowfin madtom (*Noturus flavipinnis*) with cross species amplification in *N. baileyi* Conservation Genetic Resources **4**:221-223.

Wilson, W. D., J. Hutchinson, and K. Ostrand. in press. Genetic Diversity Assessment of Wild and Refugia Texas Wild Rice (*Zizania texana*) Populations, and Endangered Plant. Aquatic Botany.

Yamamoto, S., K. Maekawa, K. Morita, P. A. Crane, and A. G. Oleinik 2014. Phylogeography of the salmonid fish, Dolly Varden *Salvelinus malma*: multiple glacial refugia in the North Pacific Rim. Zoological Science **31**:660-670.
